# Targeting of Natural Killer Cells by Rabbit Antithymocyte Globulin and Campath-1H: Similar Effects Independent of Specificity

**DOI:** 10.1371/journal.pone.0004709

**Published:** 2009-03-05

**Authors:** Diana Stauch, Annelie Dernier, Elizabeth Sarmiento Marchese, Kristina Kunert, Hans-Dieter Volk, Johann Pratschke, Katja Kotsch

**Affiliations:** 1 Institute of Medical Immunology, Charité Universitätsmedizin Berlin, Campus Mitte, Berlin, Germany; 2 Immunology Department, Hospital General Universitario Gregorio Marañon, Madrid, Spain; 3 Department of Surgery, Charité Universitätsmedizin, Campus Virchow, Berlin, Germany; University of Sheffield, United Kingdom

## Abstract

T cell depleting strategies are an integral part of immunosuppressive regimens widely used in the hematological and solid organ transplant setting. Although it is known to induce lymphocytopenia, little is known about the effects of the polyclonal rabbit antithymocyte globulin (rATG) or the monoclonal anti-CD52 antibody alemtuzumab on Natural Killer (NK) cells in detail. Here, we demonstrate that induction therapy with rATG following kidney/pancreas transplantation results in a rapid depletion of NK cells. Treatment of NK cells with rATG and alemtuzumab in vitro leads to impairment of cytotoxicity and induction of apoptosis even at a 10-fold lower concentration (0.1 µg/ml) compared with T and B cells. By generating Fc-parts of rATG and alemtuzumab we illustrate that their ligation to FcγRIII (CD16) is sufficient for the significant induction of degranulation, apoptosis and inflammatory cytokine release (FasL, TNFα and IFNγ) exclusively in CD3^−^CD56^dim^ NK cells whereas application of rATG and alemtuzumab F(ab) fragments abolishes these effects. These findings are of general importance as our data suggest that NK cells are also mediators of the clinically relevant cytokine release syndrome and that their targeting by therapeutic antibodies should be considered as they are functionally relevant for the effective clearance of opportunistic viral infections and anti-tumor activity posttransplantation.

## Introduction

Antibodies raised against certain T cell antigens are increasingly used in patients undergoing hematopoietic stem cell transplantation (HSCT) or solid organ transplantation (SOT) in order to prevent acute graft-versus-host disease (GvHD) or acute steroid-resistant graft rejection [1]. The polyclonal antithymocyte globulin (ATG) is a mixture of purified immunoglobulins M (IgM) and G (IgG) of sera derived from rabbits, horses, or goats immunized with human thymocytes or T cell lines. The most widely used preparations include rabbit ATG (rATG, IgG) which contains antibodies directed against a wide array of antigens involved in the immune response. These comprise selectin and integrin family members or immunoglobulin superfamily molecules expressed on the surface of T lymphocytes. Other cell types such as endothelial or B cells are also recognized by rATG due to shared epitopes with T cells [2], [3]. However, the key mechanism of rATG action is T cell depletion [4], [5], as it has been shown that CD3^+^ cell counts are lowered for years in patients treated with rATG [6]–[8]. Additionally, rather than T cell depletion as one of the key mechanisms, rATG has been demonstrated to affect dendritic cells [9] or to induce regulatory T cells in vitro and in vivo [10]–[12].

Alemtuzumab (Campath-1H), a fully humanized CD52-specific monoclonal antibody which profoundly depletes T, B and dendritic cells [13], [14], is also increasingly used as an immunosuppressive agent in solid organ transplantation, particularly in the setting of maintenance immunosuppression minimization protocols [15]–[17]. Additionally this monoclonal antibody has been demonstrated to be beneficial in the treatment of lymphoid malignancies and autoimmune diseases [18], [19]. However, despite the frequent use of rATG or alemtuzumab in clinical transplantation and extensive knowledge about the effects on T cells, only limited information is available about the influence of these therapeutic antibodies on Natural Killer (NK) cells. NK cells, which are part of the innate immune system, rapidly kill a wide range of hazardous pathogens such as viruses, bacteria, and parasites. They are able to kill a variety of tumor cells without prior sensitization and, through secretion of cytokines, NK cells are involved in the regulation of T and B cell-mediated immune response. In general, the lytic activity of NK cells is controlled by different activating NK receptors such as NKG2D and the natural cytotoxic receptors (collectively named NCRs, including NKp46, NKp44 and NKp30) [20]. NK cells were further shown to mediate antibody-dependent cytotoxicity (ADCC) through the FcγRIII (CD16) receptor, since anti-CD16 antibodies were able to inhibit ADCC and immune complex binding [21].

In recent years it has been shown that NK cell alloreactivity is beneficial following allo-HSCT because it mediates a graft-versus-leukemia (GvL) effect, eliminating residual malignant cells, removing host antigen-presenting cells (thereby reducing GvHD), and mediating immunity to viral pathogens directly through the cytolysis of virally infected tissues or indirectly by elaborating inflammatory cytokines, such as interferons (IFNs) [22], [23]. The antiviral capacity of NK cells is even more important, as Epstein–Barr virus (EBV) or cytomegalovirus (CBV) infections, for example, are frequent complications of prolonged immune deficiency [24], [25]. In this context, both rATG and alemtuzumab have been suggested to be associated with an elevated incidence of EBV/CMV reactivation and disease [26], [27]. In general, the influence of different immunosuppressive drugs on NK cell function is of particular interest as it has recently been demonstrated that steroids and calcineurin inhibitors limit the function of IL-2-activated NK cells [28], [29].

Given that both rATG and alemtuzumab mediate multiple immunomodulatory mechanisms in vitro and in vivo [9], [30]–[32] we sought to extend these studies by investigating the effects of these antibodies on NK cells. In summary, we demonstrated that both rATG and alemtuzumab induce rapid apoptosis in NK cells and a strong induction of inflammatory cytokines, which is exclusively mediated via the binding of the IgG1 Fc-part to the low-affinity receptor for IgG, CD16 (FcγRIII).

## Materials and Methods

### Patients

Between October 2007 and May 2008 eight simultaneous renal/pancreas patients received a non-HLA-identical allograft from deceased donors at the Departments of Surgery and Nephrology, Virchow-Clinic, Universitätsmedizin Charité, Berlin. Transplant patients received initially 1.5 mg/kg body weight i.v. Thymoglobulin (Genzyme GmbH, Neu Isenburg, Germany) starting at day 0 followed by 4 further consecutive days in combination with tacrolimus, mycophenolate mofetil and steroids. Nine patients receiving renal allografts served as controls. These patients received two dosages of basiliximab (20 mg i.v. 2 hours before reperfusion an on day 4) which has been implemented as standard therapy in our clinic for prophylaxis of rejection as well as maintenance immunosuppression consisting of tacrolimus, mycophenolate mofetil and prednisone. Blood drug concentrations of tacrolimus ranged between 8 and 10 ng/ml in both groups. All experiments using human material were approved by the Ethics Committee of the Charité-Universitätsmedizin Berlin and all patients agreed to participate and signed an informed consent. Patient demographics are summarized in Table 1.

**Table 1:** Patient demographics.

	*female/male (n)*	*LD/BD (n)*	*age (yrs) (SD)*	*1. Tx*	*re Tx*	*PRA Class I Class II*		*CI (hrs) mean (SD)*	*HLA-Mismatch (SD)*	*graft function*		*aRx//no Rx*
										early	delayed	
**ATG (n = 8)**	2/6	0/9	48±13	9	0	1/7	1/7	9.8±2.9	4.3±1.2	7	1	1/7
**non ATG (n = 9)**	7/2	0/9	52±11	9	0	1/8	1/8	11.6±3.3	2.2±1.5	9	0	0/9

LD, living donor; BD brain dead donor; PRA, panel reactive antibodies; CI, cold ischemia; aRx, acute cellular mediated rejection.

### Enrichment of NK cells and cell culture conditions

Peripheral blood from transplant patients was collected pretransplant and three times/week/patient during hospitalization. Peripheral blood mononuclear cells (PBMCs) were isolated using ficoll density centrifugation with Bicoll Separating Solution (Biochrom, Berlin, Germany). For NK cell enrichment PBMCs were isolated from de-identified healthy donor buffy coats using ficoll density centrifugation with Bicoll Separating Solution (Biochrom, Berlin, Germany) and NK cells were obtained by negative purification using the NK Cell Isolation Kit II (Miltenyi Biotech, Bergisch Gladbach, Germany) according to the manufacturer's recommendation. The purity of the NK cells (CD3^−^CD56^+^) was monitored by fluorescence-activated cell sorting (FACS) and was more than 95%. Enriched NK cells were cultured in RPMI 1640 medium supplemented with 10% heat-inactivated fetal calf serum (FCS), 1% Glutamin and antibiotics (penicillin 100 U/ml, streptomycin 100 µg/ml) in the presence of 200 IU/ml human recombinant IL-2 (Natutec, Frankfurt am Main, Germany). NK cell subsets (CD56^bright^/CD16^−^, CD56^dim^/CD16^+^) were enriched by flow cytometry (FACSAria, BD Biosciences), giving a purity of >99%. Rabbit ATG (Thymoglobulin, Genzyme, Neu Isenburg, Germany) was dissolved in sterile distilled water at a concentration of 5 mg/ml. Alemtuzumab (Campath-1H, MedacSchering, München, Germany) was delivered in a solution of 30 mg/ml. The monoclonal anti-CD3 antibody (Orthoclone OKT3, Janssen-Cilag BV, Tilburg, The Netherlands), the humanized anti-IL2Rα receptor (CD25) antibody daclizumab (Zenapax®; Hoffman La Roche, Basel, Switzerland) and a polyclonal rabbit IgG (Bethyl Laboratories, Inc., Montgomery, Texas, USA) served as controls. Varying concentrations of antibodies ranging from 0 to 100 µg/ml were added to the NK cell cultures for 18 hours or as indicated in the figure legends. All experiments using human material were approved by the Ethical Committee of the Charité-Universitätsmedizin Berlin.

### Ex vivo whole blood (WB) assay

Whole blood of volunteer donors was collected into a BD vacutainer (Becton Dickinson, NJ, USA). One ml of fresh whole blood was co-incubated with 0, 1, 10 and 50 µg/ml of intact antibody or Fc-parts of rATG and alemtuzumab for 2 hours at 37°C with 5% CO_2_. Red blood cells were lysed with lysis buffer (NH_4_Cl, 0.155 M; KHC0_3_, 0,01 M; EDTA, 1 mM). Preparation of mRNA and quantitative real-time RT-PCR for FasL, TNFα, INFγ and HPRT were performed as described below.

### Antibodies and reagents

F(ab) fragments and Fc-parts of rATG, alemtuzumab and anti-CD16 (clone 3G8, a kind gift from Dr. Ofer Mandelboim, The Hebrew University of Jerusalem, Israel) were prepared by papain digestion and purified by exclusion chromatography on protein A according to standard procedures. NK cells were analyzed by FACS using the following monoclonal antibodies (mAB): anti-CD3 FITC mAb (clone BW264/56, IgG2a), anti-CD3 PE mAb (clone BW264/56, IgG2a), anti-CD56 APC mAb (clone AF12-7H3, mouse IgG1), anti-CD16 PE (clone VEP13, mouse IgM), anti-CD8 PE (clone BW 135/80, mouse IgG2a), anti-NKp30 PE (clone AF29-4D12, mouse IgG1), anti-NKp44 PE (clone 2.29, mouse IgG1), anti-NKp46 PE (clone 9E2, mouse IgG1), anti-NKG2D PE (clone BAT221, mouse IgG1), anti-KIR3DL1 PE (clone DX9, mouse IgG1), anti-IFNγ FITC (clone 45-15, mouse IgG1) (Miltenyi Biotech, Bergisch Gladbach, Germany); anti-CD3 PeCy5 (clone UCHT1, mouse IgG1) (ImmunoTech, Marseille, France) and anti-KIR2DL1 FITC (clone 143211, IgG1) (R&D, Minneapolis, USA). Isotype controls conjugated with FITC, PE, APC and PeCy5 (Ebioscience, San Diego, USA) were used to discriminate unspecific binding. 2×10^5^ cells were incubated in PBS supplemented with 1% fetal calf serum (FCS) and stained with the appropriate antibodies for 30 min at 4°C. For intracellular IFNγ analysis expression, Brefeldin A (Sigma, St. Louis, USA) was added to the cell culture after 1 hour of incubation for further 2 hours and cells were surface-stained and fixed with freshly prepared 2% paraformaldehyde in PBS. 0.1% Saponin (Sigma, St. Louis, USA) was used for permeabilization. Cells were analyzed on a Becton Dickinson FACSCalibur® interfaced with an Apple PowerMac using Cellquest Pro software. Cell culture supernatants were analyzed for the concentration of soluble cytokines TNFα and IFNγ by Cytometric Bead Array System (CBA) from BD (Becton Dickinson, Heidelberg, Germany) according to the company's instructions.

### Analysis of apoptosis and necrosis

Apoptosis and necrosis of cells were determined by the 36 kD phospholipid-binding protein Annexin V FITC and propidium iodide (PI) according to the manufacturer's instructions (Miltenyi Biotech, Bergisch Gladbach, Germany). In brief, cells were discriminated as intact (Annexin V FITC−/PI−), apoptotic (Annexin V FITC+/PI−) or necrotic (Annexin V FITC+/PI+).

### Cytotoxicity assay

Assessment of the specific NK-cell-mediated killing was performed using the LIVE/DEAD cell-mediated cytotoxicity kit (Molecular Probes, Eugene, USA) with slight modifications. 1×10^6^ cells of the human MHC-deficient erythromyeloblastoid leukemia target cell line K562 (DSMZ, Braunschweig, Germany) were labeled with 30 nM of the membrane dye DiOC_18_(3) for 20 min and were subsequently washed with complete medium twice. A quantity of 1×10^4^ labeled K562 cells were mixed with NK cells in a 96-well plate at effector-target cell (E/T) ratios of 10∶1, 5∶1 and 2.5∶1 and were incubated for 3 hours. Afterwards propidium iodide (20 nM) was added to the cells. Samples were stored on ice until fluorescence measurements were performed. DiOC_18_(3) labeled cells were selected according to high FL1 green fluorescence intensity. Propidium iodide-positive dead target cells were identified by FL3 fluorescence and analysis was performed by dot plot. The specific killing percentage was calculated by the following equation: [% of dead (DiOc_18_(3)+PI+) target cells/(% of dead (DiOc_18_(3)+PI+) target cells+% of live (DiOc_18_(3)+PI−) target cells)]×100. The specific killing percentage was normalized by subtracting the percentage of dead target cells without killing. Every sample was prepared and analyzed in triplicate.

### Degranulation assay

CD107a lysosome-associated membrane protein-1 (LAMP-1) expression was used to measure NK cell degranulation, as previously described [33]. NK cells were incubated in the absence or presence of K562 target cells at an E/T ratio of 2∶1 for 3 hours. An anti-CD107a PeCy5 antibody (BD Pharmingen™, NJ, USA) was added to the cultures directly and Brefeldin A was added after 1 hour of incubation. Cells were then washed in PBS and stained with anti-CD56 and anti-CD3 for FACS analysis. The K562 specific NK cell degranulation was determined by subtracting the percentage of CD107a-positive cells in the cultures without K562 cells from the percentage of CD107a-positive NK cells co-incubated with K562 cells.

### Quantitative real-time RT-PCR

Total RNA from cell lysates was isolated using RNeasy® Mini Kit (Qiagen, Hilden, Germany). Real-time reverse transcriptase polymerase chain reaction (RT-PCR) for gene expression analysis of selected candidate genes was performed with the ABI PRISM 7500 Sequence Detection System (TaqMan™, Applied Biosystems, Darmstadt, Germany). All primers were designed using Primer Express software (Applied Biosystems) and validated at the Institute of Medical Immunology, Charité-Universitätsmedizin Berlin (Table S1). In general, amplification primers were designed to span the exon borders to exclude cross-reactivity with genomic DNA. The PCR reaction was performed in a final volume of 25 µl containing 1 µl cDNA, 12.5 µl Master Mix (TaqMan™ Universal PCR Master Mix, Applied Biosystems), 1 µl fluorogenic hybridization probe, 6 µl primer mix, and 5.5 µl distilled water. The amplification took place in a two-step PCR (40 cycles; 15 s denaturation step at 95°C and 1 min annealing/extension step at 60°C). Specific gene expression was normalized to the housekeeping gene hypoxanthine-guanine phosphoribosyltransferase (HPRT) given by the formula 2^−ΔCt^. The mean Ct values for the genes of interest (IFNγ, TNFα, FasL) and HPRT were calculated from double determinations. Samples were considered negative if the Ct values exceeded 40 cycles.

### Statistical analysis

Experimental and patient data are expressed as means±standard deviation (SD). Statistical analyses were performed with GraphPad Instat for Windows (version 3.06). One-way ANOVA was calculated for normally distributed data. Adjustments for multiple comparisons were done with the Tukey-Kramer Multiple Comparisons Test. For the statistical analysis of apoptosis and necrosis, cytotoxicity, degranulation and surface FACS staining rare data were used. The real-time RT-PCR data were log(10) transformed to stabilize the variance of the normally distributed data. Groups with p values less than or equal to 0.05 were considered to be statistically different.

## Results

### In vivo depletion of NK cells after ATG induction therapy following pancreas/kidney transplantation

**Figure 1:**
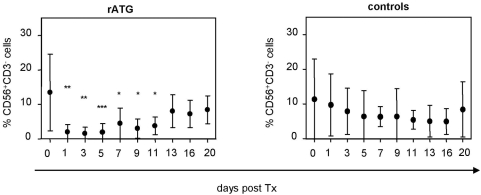
Induction therapy of rATG in simultaneous kidney/pancreas transplantation results in a significant decrease of NK cells. Patients (n = 8) initially received 1.5 mg/kg body weight i.v. rATG (Thymoglobulin, Genzyme GmbH, Neu Isenburg, Germany) starting at day 0 followed by 4 further consecutive days posttransplantation in combination with tacrolimus, mycophenolate mofetil and steroids. We further enrolled nine patients who received a renal allograft as a control group. Control patients received two dosages of basiliximab (20 mg i.v., day 0 and day 4). Asterisks denote significant differences compared to pretransplant levels of CD3^−^CD56^+^ NK cells.

Patients treated with rATG were found to demonstrate a significant decrease of CD3^−^CD56^+^ NK cells within the peripheral blood lymphocytes following the first day posttransplantation (13.4±10.6% pretransplantation versus 2.0±2.1% first day posttransplantation, p<0.01) and this effect is persisting until day 11. In contrast control patients illustrate nearly none or only a marginal decrease of NK cells after the application of basiliximab (Figure 1). Although NK cell numbers did not reach the pretransplant level at days 20 of observation, frequencies of NK cells between rATG treated and control patients showed comparable levels at this time point.

### rATG and alemtuzumab recognize the surface antigen CD16 on NK cells

**Figure 2:**
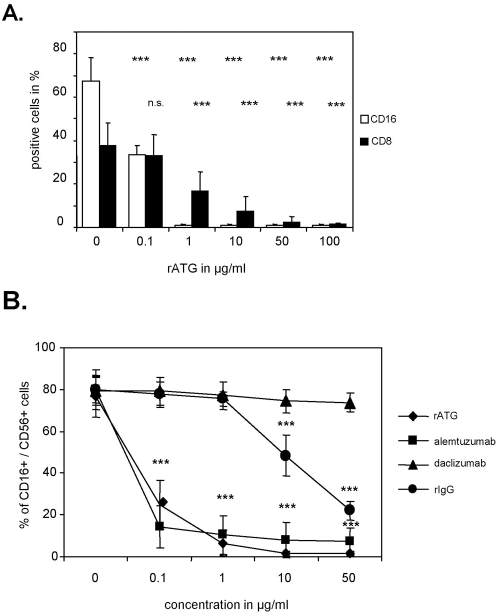
Rabbit ATG and alemtuzumab target surface expression of CD16 on NK cells. Human NK cells cultured with IL-2 (200 IU/ml) and different concentrations of rATG, alemtuzumab or control antibodies such as rIgG and daclizumab (18 hours) were harvested, washed and stained for CD3, CD56, CD8 and CD16. (A) The figure illustrates the average percentages of CD16 and CD8 staining on CD3^−^CD56^+^ NK cells after co-incubation with varying concentrations of rATG. Results are displayed as means±SD (n = 5); asterisks (*) denote significant differences compared to untreated controls: ***p<0.001. (B) The mean percentage of CD16 on CD3^−^CD56^+^ NK incubated with different concentrations of rATG, alemtuzumab, daclizumab and rIgG is shown. Results are displayed as means±SD of five independent experiments. Asterisks (*) indicate values that showed significantly less CD16 expression compared to untreated controls: ***p<0.001.

NK cells derived from CD34^+^ hematopoietic stem cells undergo differentiation via NK cell precursors in the bone marrow through acquisition of functional surface receptors [34]. Therefore, certain NK cell specific receptors should not be targeted by rATG. As expected we could not detect any influence of rATG on the surface expression of the activating cytotoxicity receptors NKp30, NKp44, NKp46, NKG2D and killer-cell immunoglobulin-like receptors (KIRs) including KIR2DL1 and KIR3DL1 (data not shown). In contrast we confirmed previous data illustrating that rATG targets CD8 and CD16 and this effect was dose-dependent (Figure 2A) [35]. Targeting of CD16 was observed in the presence of 0.1 µg rATG (67%±10.8% versus 33.6%±4.1%, p<0.001), and CD16 was further decreased to 1.2%±0.6% at a concentration of 1 µg/ml rATG. In contrast, targeting of CD8 antigen required higher concentrations of rATG (e.g. 10 µg, 38.6±11.9% versus 9.6±4.3%, p<0.001). Whereas both rATG and alemtuzumab resulted in a significant and dose-dependent reduction of CD16 surface expression on CD56^+^ NK cells at a low dose concentration of 0.1 µg/ml, no CD16 downmodulation could be observed for daclizumab, an anti-IL-2Rα (CD25) humanized antibody similar to alemtuzumab (Figure 2B). In contrast, higher concentrations of rabbit IgG (rIgG) led to a decrease of CD16 (e.g. 10 µg, 80%±6.3% versus 48.4%±9.7% and 50 µg/ml 22.1%±4.3%, p<0.001).

### Both rATG and alemtuzumab affect effector functions of peripheral blood CD3^−^/CD56^dim^ NK cells

**Figure 3:**
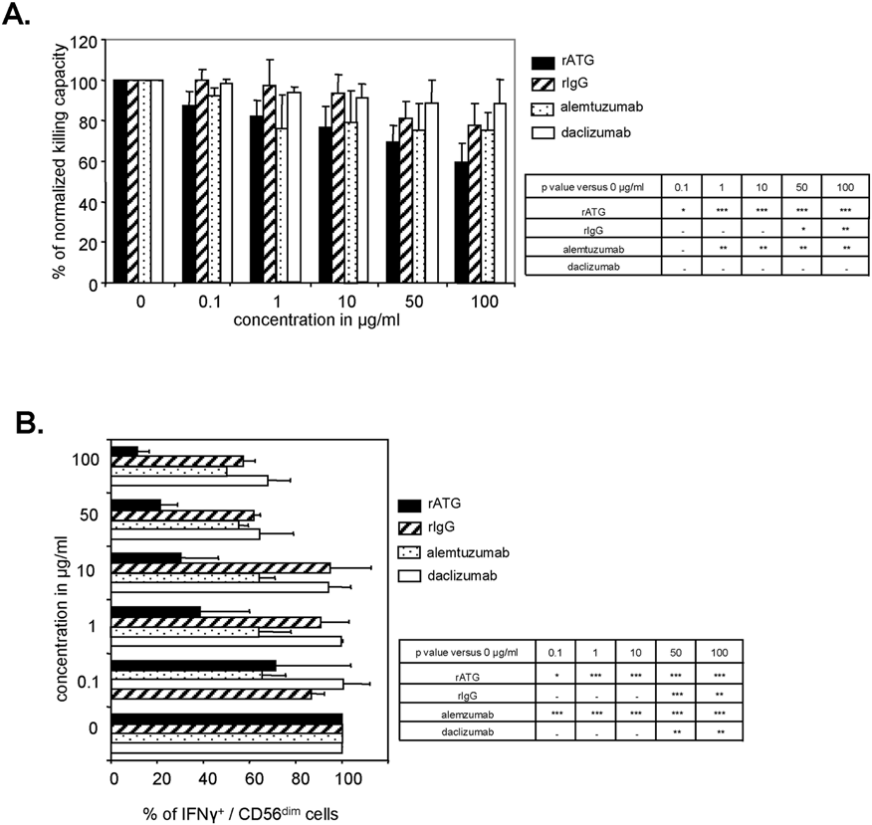
Rabbit ATG and alemtuzumab decrease effector mechanisms of peripheral blood CD3^−^CD56^dim^ NK cells. (A) Dose-dependent decrease of NK cell cytotoxicity after 18 hours pretreatment with rATG, alemtuzumab, daclizumab and rIgG (0–100 µg). Killing assay for viable and antibody-depleted NK cells was performed using the target cell line K562 (E/T ratio was 10∶1). Analysis of six independent experiments was performed by flow cytometry. Values demonstrate results normalized to untreated cells, which were set at 100%. Asterisks (*) indicate values that showed significantly less cytotoxicity compared to control: *p<0.05, **p<0.01, ***p<0.001. (B) K562 cells (E/T ratio 2∶1) were added to NK cells cultured for 18 hours with IL-2 (200 IU/ml) and varying concentrations of rATG, alemtuzumab, daclizumab and rIgG. Cells were harvested, stained and analysis was performed for CD3^−^CD56^dim^IFNγ^+^ NK cells. Values demonstrate the mean of INFγ^+^/CD56^dim^ NK cells normalized to untreated cells, which were set at 100%. Results are displayed as means±SD for 6 independent experiments. Asterisks (*) indicate values that showed significantly less IFNγ expression compared to untreated controls: *p<0.05, **p<0.01, ***p<0.001.

Alemtuzumab is also used as induction therapy in clinical transplantation, inducing profound and prolonged lymphopenia. As recent reports suggest that alemtuzumab may enhance lymphocyte apoptosis in vitro in the absence of complement or immune effector cells [36], we ascertained its effects on NK cells. NK cells were isolated from healthy blood donors and cultured in the presence of human recombinant IL-2 (200 IU/ml) with increasing concentrations of antibodies overnight (18 hours). Treatment of NK cells with rATG resulted in a dose-dependent (0.1–100 µg/ml) decrease of NK cytotoxicity directed against the target cell line K562, as illustrated in Figure 3A (e.g. 1 µg/ml rATG resulted in 82.0%±8.1% NK cell cytotoxicity versus 100% cytotoxicity of untreated controls, p<0.001) whereas control rIgG affected NK cytotoxicity at higher concentrations (50 µg/ml rIgG resulted in 81.1%±11.1% versus 100% cytotoxicity of untreated controls, p<0.05). Co-incubation of NK cells with alemtuzumab also led to an impairment of cytotoxicity (1 µg/ml alemtuzumab resulted in 75.9%±16.6% versus 100% cytotoxicity of untreated controls, p<0.01) whereas daclizumab did not (Figure 3A). Based on their CD56 receptor expression density human NK cells can be distinguished as CD56^dim^ or CD56^bright^ NK cells. In peripheral blood the majority (>90%) are CD56^dim^ demonstrating high expression of FcγRIII (CD16), while the remaining 10% are CD56^bright^ NK cells characterized by almost no or dim expression of CD16 [37]. As cytotoxicity was sharply targeted by rATG, we analyzed the intracellular protein expression of IFNγ as an effector molecule in NK cells after co-incubation with K562 cells and detected a dose-dependent impairment exclusively in CD56^dim^ NK cells, whereas no influence was observed in CD56^bright^ NK cells (Figure 3B, data not shown). Again, a significant decrease of IFNγ was observed even at a low dose concentration of 0.1 µg/ml rATG (71.2%±35.5% versus 100%, p<0.05) or alemtuzumab (65.4%±10.1% versus 100%, p<0.001). Interestingly, higher dosages such as 50 µg/ml of rIgG (61.5%±3.0% versus 100%, p<0.001) or daclizumab (64.6%±14.5% versus 100%, p<0.01) further led to decreased IFNγ expression (Figure 3B).

**Figure 4:**
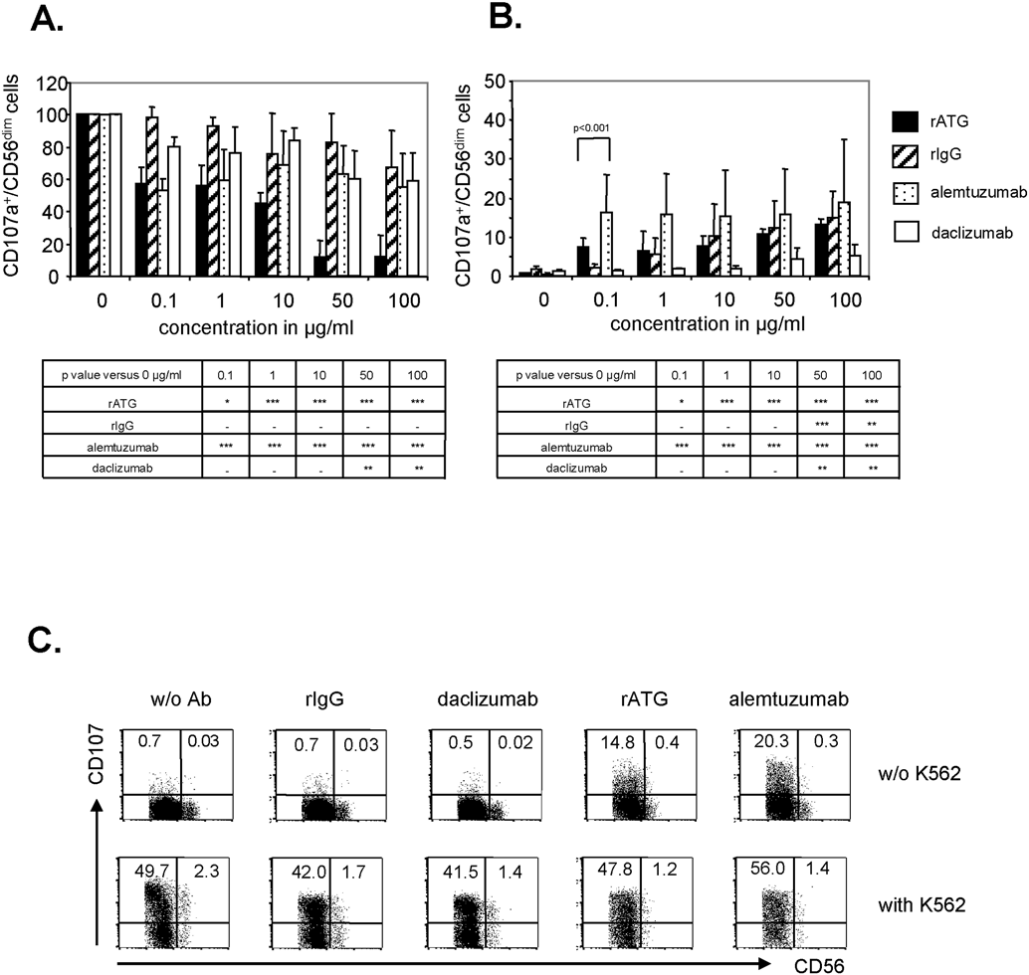
Rabbit ATG and alemtuzumab influence the degranulation of peripheral blood CD3^−^CD56^dim^ NK cells. (A) K562 cells (E/T ratio 2∶1) and CD107a antibody were added to NK cells cultured with IL-2 (200 IU/ml) and varying concentrations of rATG, alemtuzumab, daclizumab and rIgG. NK cells were harvested and stained for CD3 and CD56. For analysis the percentage of CD107a^+^ NK cells of controls (without K562) was subtracted from the CD107a^+^ NK cells co-incubated with K562 cells. Values demonstrate CD107a expression on CD56^dim^ NK cells normalized to untreated cells, which were set at 100%. Results are displayed as means±SD for 5 independent experiments. Asterisks (*) indicate values that showed significantly less degranulation compared to untreated controls: *p<0.05, ** p<0.01, *** p<0.001. (B) A degranulation assay was performed (A) without the addition of K562 cells. Results are displayed as the mean of CD107a^+^ cells on CD56^dim^ NK cells±SD (n = 5). Asterisks (*) indicate values that showed significantly higher degranulation compared to untreated controls: *p<0.05, ** p<0.01, *** p<0.001. (C) Representative FACS dot plots of CD56^+^CD3^−^ NK cells stained for CD107a. Treatment with 0.1 µg/ml rATG and alemtuzumab produced a higher induction of CD107a in CD56^dim^ NK cells compared to 0.1 µg/ml daclizumab and rIgG.

When we analyzed the degranulation capacity of NK cells preincubated with rATG by the measurement of CD107a, a marker significantly upregulated on the surface of NK cells following stimulation with MHC-devoid targets, we detected a significant impairment even at a low concentration of 0.1 µg/ml rATG, and this was observed exclusively in CD56^dim^ NK cells (56.8%±11.6% versus 100%, p<0.05, Figure 4A). As was seen with rATG, a significant decrease of degranulation capacity of CD56^dim^ NK cells after pre-incubation with alemtuzumab was detected, even at a concentration of 0.1 µg/ml (52.5%±7.8% versus 100%, p<0.001, Figure 4A), in contrast to control antibodies (rIgG, daclizumab), which exhibit induction of degranulation at higher concentrations (50 µg/ml–100 µg/ml). In contrast to rATG, no dose-dependent effect was observed for alemtuzumab (Figure 4A). Interestingly, both rATG and alemtuzumab induced degranulation of NK cells without additional sensitization by adding K562 cells. In comparison with rATG a stronger CD107a induction in NK cells with alemtuzumab was detected (e.g. 0.1 µg/ml alemtuzumab 18.3%±6.6% versus 0.1 µg/ml rATG 7.3%±3.0%, p<0.001, Figure 4B,C). In contrast, induction of degranulation by rIgG and daclizumab was dose-dependent, while the latter antibody showed only moderate degranulation induction on NK cells (5.3%±2.8% degranulation, 100 µg/ml).

### Induction of apoptosis in CD3^−^/CD56^dim^ NK cells

**Figure 5:**
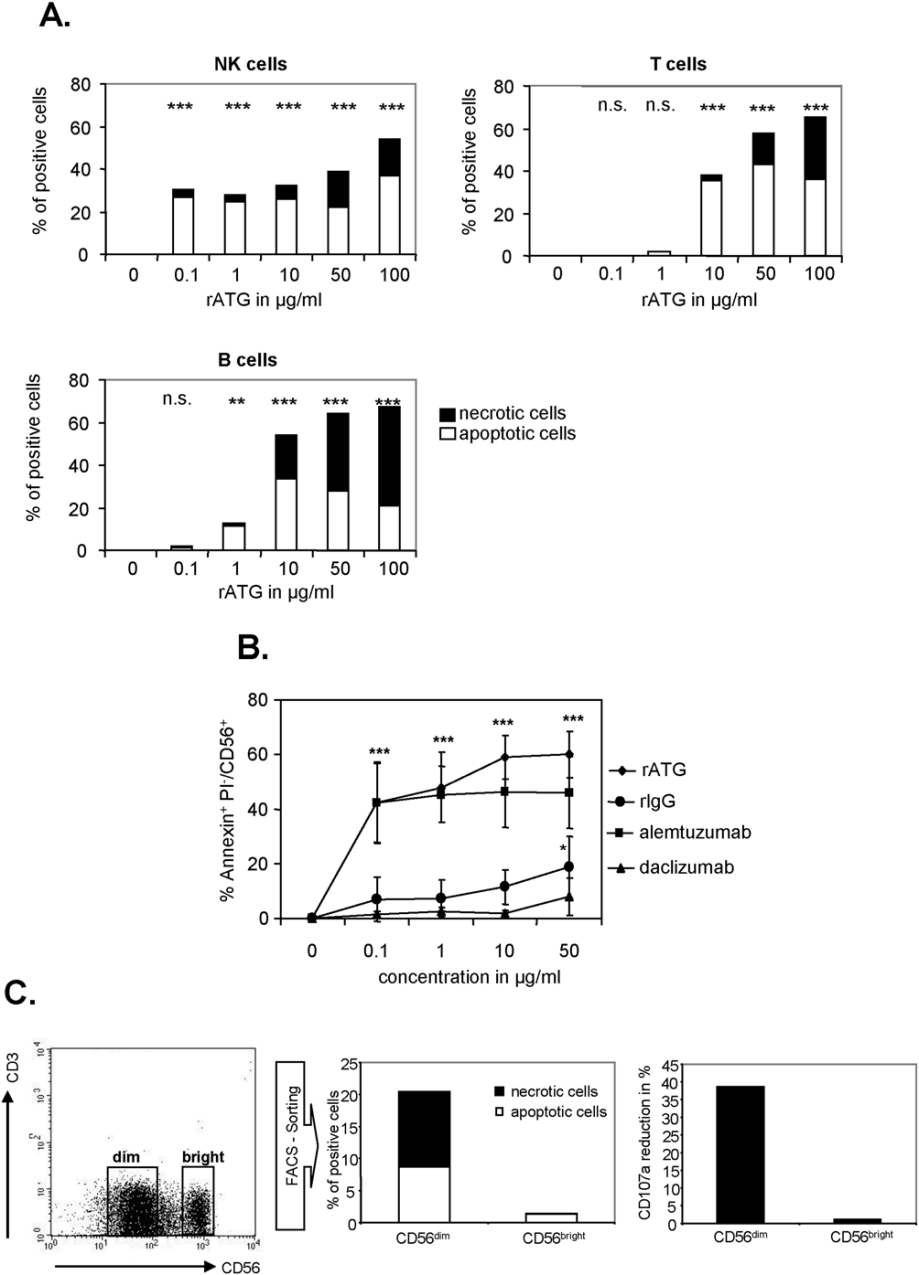
Induction of apoptosis and necrosis in CD3^−^CD56^dim^ NK cells after co-incubation with rATG or alemtuzumab. (A) Magnetically isolated NK, T and B cells from healthy volunteer blood donors were treated with different concentrations of rATG for 18 hours. Annexin V^+^ and PI^−^ cells were shown as apoptotic cells and Annexin V^+^ and PI^+^ cells as necrotic cells. Rabbit ATG induced apoptosis and necrosis of NK cells at lower concentrations (0.1 µg) compared with T and B cells. Values demonstrate the results normalized to untreated cells and are displayed as means of five independent experiments. Asterisks (*) indicate significant values: **p<0.01, ***p<0.001. (B) NK cells were cultured with IL-2 (200 IU/ml) and different concentrations of rATG, alemtuzumab, daclizumab and rIgG for 18 hours. Both rATG and alemtuzumab led to a rapid and significant induction of apoptosis of NK cells even at the low concentration of 0.1 µg/ml. In contrast, rIgG resulted in NK cell apoptosis at higher concentrations (e.g. 50 µg/ml). Values demonstrate the results normalized to untreated cells and are displayed as means of five independent experiments. Asterisks (*) indicate significant values compared to untreated controls: *p<0.05, ***p<0.001. (C) CD56^+^CD3^−^ NK cells were FACS-sorted into CD56^dim^ and CD56^bright^ subpopulations and cultured with IL2 (200 IU) and 1 µg/ml rATG for 18 hours. Determination of apoptotic and necrotic cells was performed as for (A). Rabbit ATG induced apoptosis and necrosis exclusively in CD56^dim^ NK cells (left-hand bar). A degranulation assay with K562 cells was performed and the mean reduction of CD107a staining after treatment of the sorted populations with 1 µg/ml rATG is illustrated in the right-hand graph (n = 2). Values demonstrate the results normalized to untreated cells and are displayed as means of two independent experiments.

It has already been demonstrated that rATG induces apoptosis in NK cells [30]. We could confirm these observations by showing that even low concentrations of rATG (0.1 µg/ml) resulted in enhanced apoptosis and necrosis (30.3±10.3, p<0.001, Figure 5A) and this effect was even more intensified in NK cells compared to T or B cells. However, higher concentrations of rATG (10–100 µg/ml) led to an increased rate of apoptosis and necrosis in T and B cells, whereas the rate of necrosis and apoptosis in NK cells could not be increased (Figure 5A). Next we investigated the induction of apoptosis following alemtuzumab pretreatment and also detected a significant induction of apoptosis comparable to rATG at a concentration of 0.1 µg/ml (42.3±16.6, p<0.001, Figure 5B). Induction of apoptosis was also detected with rIgG at a concentration of 50 µg/ml. In order to verify the results observed in the bulk NK cell population we sorted CD56^dim^ and CD56^bright^ NK cells with a purity greater than 99% for each cell fraction. By analyzing FACS sorted CD56^dim^ and CD56^bright^ NK cells separately the main induction of necrosis/apoptosis was observed in the CD56^dim^ subset (Figure 5C). Again, an impairment of CD107a expression was also exclusively confirmed for CD56^dim^ NK cells.

### Induction of FasL, TNFα and IFNγ in NK cells after treatment with rATG and alemtuzumab

**Figure 6:**
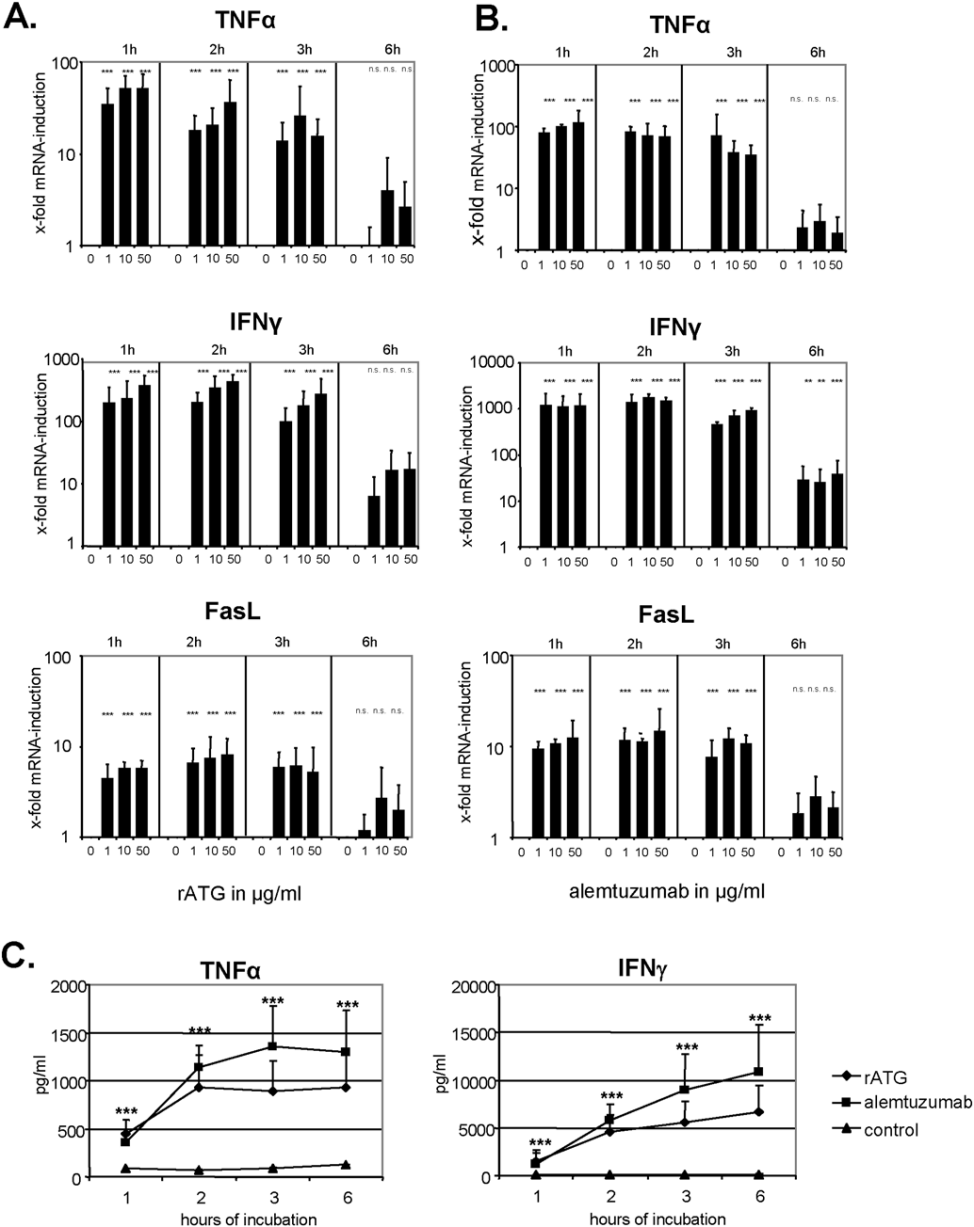
Rabbit ATG and alemtuzumab increase FasL, TNFα and IFNγ mRNA in NK cells. (A) IL-2 (200 IU/ml) pre-activated NK cells cultured in the presence of rATG were analyzed for FasL, TNFα and IFNγ mRNA after 1, 2, 3 and 6 hours of co-culture. rATG induced a rapid and dose-dependent induction of FasL, TNFα and IFNγ mRNA in NK cells which decreased after 6 hours of co-incubation. (B) Similar to rATG, the application of alemtuzumab results in a rapid FasL, TNFα and IFNγ mRNA induction within the first hour of co-incubation. Values demonstrate the results relativized to untreated controls (2^−ΔΔct^) and are displayed as means of six independent experiments. Asterisks (*) indicate significant values compared to untreated controls: **p<0.01, ***p<0.001. (C) Induction of cytokines by rATG and alemtuzumab (10 µg/ml) was further confirmed for TNFα and IFNγ at the protein level, illustrating a significant induction over time. Asterisks (*) indicate significant values: ***p<0.001 compared to untreated controls.

The stimulation of CD16 (FcγRIII) on NK cells can initiate autocrine programmed cell death by the up-regulation of, for example, Fas ligand (FasL) [38]. Whereas freshly isolated NK cells did not constitutively express FasL mRNA, incubation with rATG resulted in a rapid and dose-dependent mRNA induction within the first hour (p<0.001) which decreased after 6 hours of co-culture (Figure 6A). Furthermore it has been demonstrated that anti-T-cell therapy results in the cytokine release syndrome early after administration [39], [40]. Similar to the induction of FasL mRNA, we detected a dose-dependent increase of TNFα and IFNγ mRNA in NK cells within the first hour of co-incubation with rATG (p<0.001, Figure 6A). Additionally, the same induction of FasL, TNFα and IFNγ mRNA was observed for alemtuzumab-treated NK cells (p<0.001, respectively), although the induction of IFNγ and TNFα mRNA was more intense compared to rATG-pretreated NK cells (Figure 6B). Analysis of the control antibodies rIgG and daclizumab showed that both induced a cytokine induction at higher concentrations (10–50 µg/ml), which is clearly below the induction profile of rATG and alemtuzumab (Figure S1). We confirmed the rapid and significant induction of TNFα and IFNγ at the protein level in cell culture supernatants (p<0.001, respectively, Figure 6C), further demonstrating that alemtuzumab resulted in higher cytokine expression levels compared to rATG.

### The Fc-part of rATG and alemtuzumab is sufficient to induce cytokine release, apoptosis and degranulation via FcγRIII ligation

**Figure 7:**
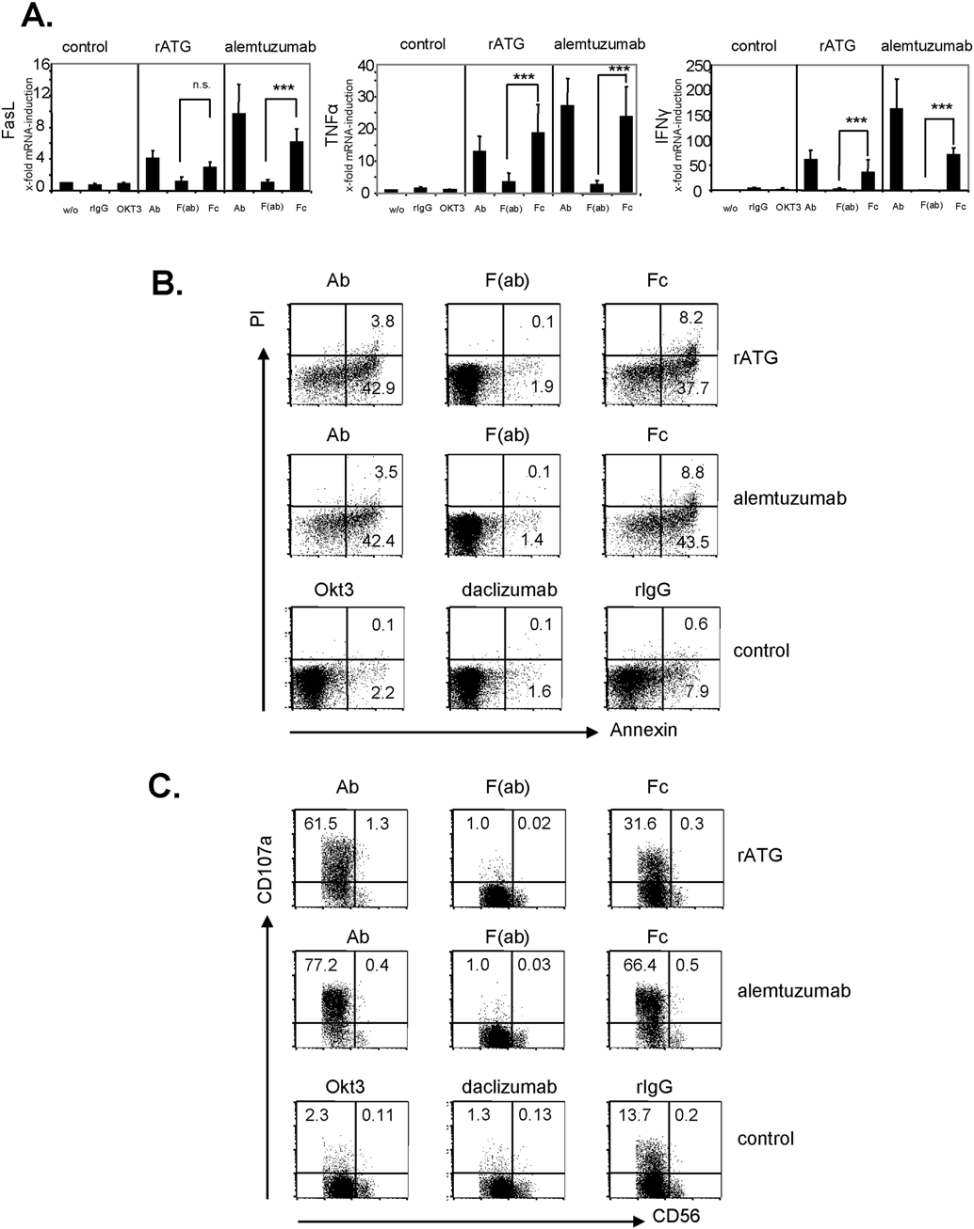
The IgG1 Fc-part of rATG and alemtuzumab is sufficient to induce enhanced cytokine expression, apoptosis and degranulation. IL-2 (200 IU/ml) preactivated NK cells were incubated for 1 hour with either 1 µg/ml whole IgG antibodies, Fc-parts or F(ab) fragments of rATG or alemtuzumab. Cells treated with anti-CD3 (OKT3, IgG2a) or rabbit IgG served as controls. (A) Values for FasL, TNFα and IFNγ mRNA demonstrate the results relativized to untreated controls (2^−ΔΔct^) and are displayed as means±SD (n = 5): ***p>0.001. (B) Preactivated NK cells with IL-2 (200 IU/ml) were incubated with either 10 µg/ml intact antibody, Fc-parts or F(ab) fragments of rATG and alemtuzumab, or OKT3 for 1 hour. The antibodies daclizumab and rIgG served as controls. FACS dot plots illustrate staining for Annexin V and PI of treated CD56^+^CD3^−^ NK cells. One representative of four independent experiments is shown. (C) Preactivated NK cells with IL-2 (200 IU/ml) were incubated with 10 µg/ml antibody preparations and anti-CD107a mAb for 3 hours. FACS dot plots illustrate staining for CD107a of CD56^+^CD3^−^ cells. One representative of four independent experiments is shown.

**Figure 8:**
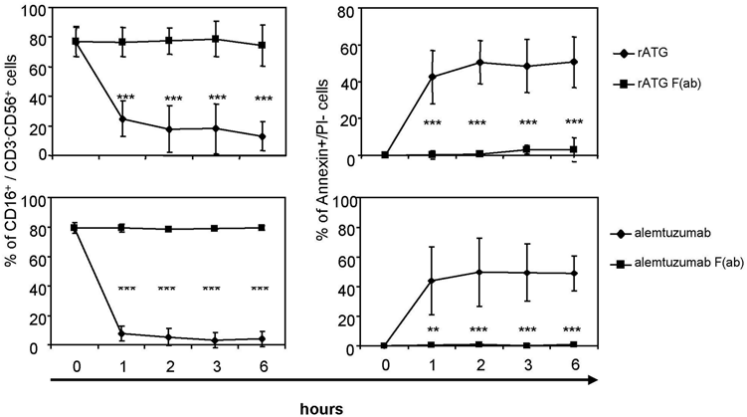
Application of F(ab) rATG and alemtuzumab fragments abolishes apoptosis and targeting of CD16 on CD56^+^CD16^+^ NK cells. NK cells were treated either with 1 µg/ml rATG or alemtuzumab or with 1 µg/ml F(ab) fragments of rATG or alemtuzumab for 1, 2, 3 and 6 hours. Controls remained untreated. Values of CD56^+^CD16^+^ NK cells are displayed as means±SD (n = 5); asterisks (*) indicate significant values compared to controls (***p<0.001, left panel). F(ab) fragments of rATG or alemtuzumab did not induce apoptosis and did not target CD16. Annexin V^+^/PI^−^ CD56^+^CD3^−^ cells were normalized to untreated cells. Values are displayed as means±SD of five independent experiments. P values are related to the rATG/alemtuzumab treatment: ***p<0.001.

In order to investigate the influence of the unspecific binding of the Fc-part to FcγRIII, we generated F(ab) fragments and Fc-parts of a CD16 blocking antibody (clone 3G8), rATG and alemtuzumab. Blocking FcγRIII on NK cells by F(ab) fragments of an anti-CD16 resulted in a significant inhibition of FasL (p<0.001), TNFα (p<0.05) and IFNγ (p<0.001) mRNA induction. Additionally, the application of F(ab) fragments of either rATG or alemtuzumab further did not cause FasL, TNFα and IFNγ mRNA induction (Figure S2). Next, we tested generated Fc-parts and could illustrate that the application of rATG and alemtuzumab Fc-parts only is sufficient to induce a significant cytokine induction and apoptosis in NK cells compared with F(ab) fragments. This induction showed a similar expression level compared with intact IgG antibodies. In contrast, the application of control rIgG, a monoclonal anti-CD3 antibody (OKT3, IgG2a isotype) or daclizumab (Figure S1, 1 µg/ml for 1 hour) did not lead to induced cytokine levels and increased apoptosis (Figure 7A,B). Moreover, CD16 ligation by rATG and alemtuzumab Fc-parts resulted in degranulation of NK cells further emphasizing that antigen-specific crosslinking of antibodies is not necessary for the observed effector effects (Figure 7C). After the addition of rATG or alemtuzumab F(ab) fragments, targeting of CD16 on CD56^+^CD16^+^ cells and induction of NK cell apoptosis was abolished compared to the application of intact IgG antibodies (p<0.001, Figure 8). Thus, our results clearly illustrate that FcγRIII ligation with the Fc-part of rATG or alemtuzumab is sufficient to induce NK cell apoptosis and cytokine release and that this effect is independent of antibody specificity.

**Figure 9:**
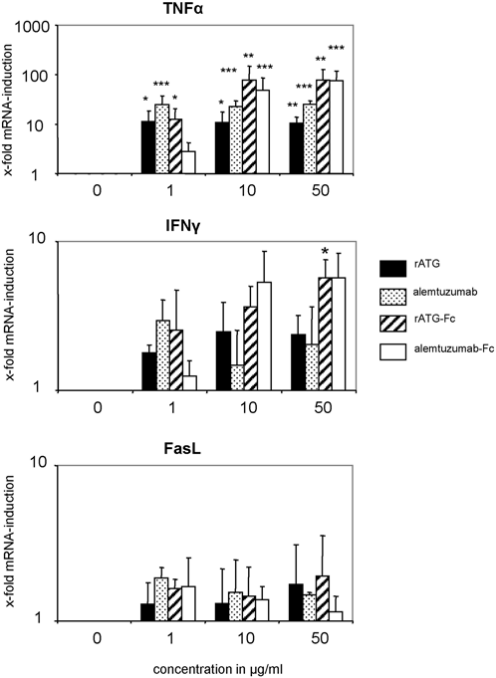
The Fc-part of rATG and alemtuzumab is sufficient to induce cytokine release in whole blood samples. Whole blood samples were treated for 2 hours with 1, 10 and 50 µg/ml of intact antibody or Fc-parts of rATG and alemtuzumab. After red blood cell lysis induction of TNFα, IFNγ and FasL mRNA was observed. Values demonstrate the results relativized to untreated controls (2^−ΔΔct^) and are displayed as means of four independent experiments. Asterisks (*) indicate significant values compared to untreated controls: * p<0.05, **p<0.01, ***p<0.001.

In order to ascertain whether high concentrations of serum immunoglobulins in whole blood may block the Fc-part-mediated cytokine release of rATG or alemtuzumab, we performed stimulation experiments in whole blood. Our results with co-incubation of whole blood with intact antibody or Fc-parts demonstrate that CD16 ligation is sufficient for TNFα, IFNγ and FasL induction in this experimental setting (Figure 9).

## Discussion

Rather than T cell depletion rATG mediates complement-related lysis or activation-associated apoptosis via the induction of Fas and FasL [41] and prevention of memory T cell migration [3], [4]. Recent studies illustrate that the therapeutic effect of rATG might be due to the generation of regulatory T cells and polarization of monocyte-derived dendritic cells towards tolerogenic dendritic cells [10], [42]. Although alemtuzumab has not been able to induce allograft tolerance as was initially hoped, it is assumed that both complement and non-complement-mediated mechanisms are similarly responsible for alemtuzumab-mediated killing of T cells. Recent reports suggest that alemtuzumab may enhance lymphocyte apoptosis in vitro in the absence of complement or immune effector cells, leading to cell death through a nonclassical caspase-independent pathway [43]. In addition it has been reported that alemtuzumab can indeed act through immunological mechanisms, such as complement-mediated (CDC) and/or ADCC by virtue of its IgG Fc region [44]. Despite the frequent use of rATG or alemtuzumab in clinical trials, detailed mechanistic studies to elucidate specific killing pathways in various lymphocyte subsets were still missing.

By analyzing patients after ATG induction therapy, treatment with rATG was shown to result in a significant decrease of CD3^−^CD56^+^ NK cells within peripheral blood lymphocytes following the first day posttransplantation. Although it has been mentioned that NK cell numbers are significantly higher in rATG treated liver transplanted patients compared with antibody-free treated patients in the long-term phase [45], our data indicate a normalization of NK cell frequencies starting at day 11 posttransplantation (Figure 1). In order to ascertain the in vitro effects on NK cells, we confirmed that surface expression of CD16 and CD8 is affected by low dose concentrations of rATG (0.1 µg/ml), and a similar observation was made for CD16 after treatment of NK cells with alemtuzumab (Figure 2A,B). We further showed that preincubation of rATG and alemtuzumab resulted in decreased effector mechanisms in NK cells, including cytotoxicity, degranulation and intracellular IFNγ production, exclusively in CD3^−^CD56^dim^ cells (Figure 3A,B, 4A,C). Interestingly both antibodies led to degranulation of NK cells even in the absence of a sensitizing target, illustrating that CD56^dim^ NK cells were more affected by alemtuzumab than by rATG (Figure 4B,C). Our results are in contrast of a recent publication [46], describing that the incubation of PBMCs with 10 µg/ml alemtuzumab without target cells did not increase CD107a expression. Moreover the same authors observed enhanced degranulation capacity of NK cells after the infusion with rituximab (anti-CD20, IgG1) in non-Hodgkin's lymphoma patients and attributed the observed anti-lymphoma effect to ADCC mediated by NK cells.

It has been shown that rATG induces apoptosis and necrosis in NK cells even at a low dose concentration (0.1 µg/ml) [30]. We could confirm this observation and further demonstrate that this induction is exclusively restricted to CD56^dim^ cells, whereas CD56^high^ cells remain unaffected. In comparison, an induction of apoptosis in B cells was observed at 1 µg/ml whereas higher concentrations in T cells were needed (10 µg/ml) (Figure 5A,B). The induction of apoptosis in T cells may differ as higher concentrations are necessary for apoptosis induction in resting T cells [41]. Additionally, the application of 0.1 µg/ml alemtuzumab also induced apoptosis in NK cells to a similar extent compared to rATG (p<0.001, respectively, antibody treated versus control, Figure 5C).

Patients treated with rATG or alemtuzumab may experience symptoms of cytokine release syndrome, reflected by elevated serum levels of TNFα, IFNγ or IL-6 [47], [48]. In the context of alemtuzumab it was demonstrated more than a decade ago in vitro that the induced cytokine release appears to be a consequence of IgG1-dependent CD16 ligation on NK cells [38]. Upon CD16-mediated activation, NK cells secrete cytokines, mediate ADCC and may undergo apoptosis as a consequence of FasL-induced cell death. We could demonstrate a rapid and significant induction of FasL, TNFα and IFNγ mRNA in NK cells within the first hour of preincubation with rATG and alemtuzumab, which was further confirmed at the protein level (p<0.001, Figure 6). By incubating NK cells with F(ab) fragments of these antibodies mRNA cytokine and apoptosis induction was significantly inhibited (Figure 7A,B). It has been also reported, that both antibody specificity and the isotype are responsible for the cytokine release [38]. In contrast to previous data our results show that the Fc-part of rATG and alemtuzumab is sufficient not only for the induction of inflammatory cytokines, but also for the induction of apoptosis and degranulation in NK cells (Figure 7).

The cytokines TNFα, IFNγ and FasL are induced after co-incubating whole blood with intact antibody or Fc-parts of rATG and alemtuzumab (Figure 9), suggesting that high concentrations of immunoglobulins in patients' sera may not block the induction of cytokine induction in vivo. However, it has to be considered in the experimental setting that cells other than NK cells are positive for CD16 (e.g. monocytes) [49], which might also contribute to enhanced cytokine induction. This might explain the different expression levels for TNFα and IFNγ observed in NK cell assays and whole blood assays. Additionally targeting of CD56^+^CD16^+^ NK cells and induction of apoptosis was abolished by applying F(ab) fragments, corroborating the importance of the Fc-part of both antibodies (Figure 8). In this context, it is established that cross-linking of Fcγ receptors is required for IgG-mediated cell activation. Since the Fc-portion is composed of two identical polypeptide chains that are related to each other by a two-fold axis of symmetry, each IgG molecule may potentially bind up to two Fcγ receptors and initiate cellular responses even in the absence of a specific antigen [50]. Although it is still discussed that the application of OKT3 is associated with cytokine release [51], our data suggest that in contrast to rATG and alemtuzumab the application of OKT3 does not result in NK mediated induction of cytokines (Figure 7).

Our results illustrate that independent of antibody specificity, rATG and alemtuzumab affect the effector functions of NK cells by the ligation of CD16 via their Fc-part. In the clinical setting peak serum levels of rATG after induction therapy in vivo range between 60–100 µg/ml for 5–7 days of treatment [47], [52] and the presence of alemtuzumab after a total dosage of 100 mg was shown to be still detectable after 28 days of treatment (1 µg/ml) in patient sera [53]. As induction of apoptosis and cytokines in NK cells was observed at a low dose concentration of 0.1 µg/ml rATG and alemtuzumab in vitro, we suggest that targeting of NK cells by these antibodies in vivo might occur even at lower concentrations as currently used in the clinic. Furthermore the increased FasL expression may enhance apoptosis in a self-sustaining loop as CD16-induced up-regulation of FasL expands the capacity of NK cells to mediate autocrine killing through Fas/FasL interactions [38].

We assume that observed differences in the potency of degranulation induction or cytokine production (Figures 4C,6) in NK cells might be due to different binding affinities of the Fc-parts as alemtuzumab is a fully humanized antibody compared with rATG although both are of IgG1 specificity. We further included rIgG as control for rATG, demonstrating that rIgG applied at higher concentrations can also result in a downmodulation of CD16 whereas no effect was observed for daclizumab. In contrast to rATG and alemtuzumab both rIgG and daclizumab resulted in an impairment of NK cell killing capacity, IFNγ production and induction of degranulation and cytokines only at higher concentrations (10–100 µg/ml). We conclude that rIgG contains various subclasses (IgG1–IgG3) and therefore displays lower specificity for CD16, in contrast to rATG, which consists of enriched IgG1 fractions. As the effector functions of antibodies are dependent on appropriate glycosylation of the antibody's Fc-region and their affinity to FcγR [54], we speculate that this aspect may be relevant for the different effector functions observed for alemtuzumab and daclizumab. Although daclizumab has been well characterized with respect to its glycosylation pattern, information about alemtuzumab is limited [55].

In summary, we demonstrated that the Fc-part of rabbit or humanized antibodies in contrast to murine Fc-parts (e.g. OKT3) is relevant and sufficient for FcγRIII mediated effects such as cytokine release, degranulation and apoptosis of NK cells. As NK cells are functionally relevant for the effective clearance of opportunistic viral infections and anti-tumor activity this should be considered in defining the optimal treatment dosage in clinical settings and for the generation of therapeutic antibodies in the future.

## Supporting Information

Table S1 (0.03 MB DOC)

Figure S1 (0.18 MB DOC)

Figure S2 (0.04 MB DOC)
