# Supplementary material for: Targeting of Natural Killer Cells by Rabbit Antithymocyte Globulin and Campath-1H: Similar Effects Independent of Specificity
Source: PLoS One. 2009 Mar 5;4(3):e4709. doi: 10.1371/journal.pone.0004709 (PMC2651595; doi:10.1371/journal.pone.0004709)
Supplement: Table S1 — (0.03 MB DOC) [file pone.0004709.s001.doc]

**Stauch et al.**

**Targeting of Natural Killer cells by rabbit antithymocyte globulin and Campath-1H: similar effects independent of specificity**

**Table S 1**

Primer sequences for real-time RT-PCR

| *Gene* | *Forward primer* | *Reverse Primer* | *Probe* |
| --- | --- | --- | --- |
| *IFNγ* | 5´caggtattcagatgtagcggataa3´ | 5´aggagacaatttggctctgcatt3´ | 5´tttctgtcactctcctctttccaattcttcaaa3´ |
| *TNFα* | 5´tctcgaaccccgagtgacaa3´ | 5´tcagccactggagctgcc3´ | 5´tgtagcccatgttgtagcaaaccctcaagc3´ |
| *FASL* | 5´atgcacacagcatcatctttgg3´ | 5´atgggccactttcctcagct3´ | 5´aagcaaataggccaccccagtccacc3´ |
| *HPRT* | 5´agtctggcttatatccaacacttcg3´ | 5´gactttgctttccttggtcagg3´ | 5´ tttcaccagcaagcttgcgaccttga 3´ |
