# Supplementary material for: Targeting of Natural Killer Cells by Rabbit Antithymocyte Globulin and Campath-1H: Similar Effects Independent of Specificity
Source: PLoS One. 2009 Mar 5;4(3):e4709. doi: 10.1371/journal.pone.0004709 (PMC2651595; doi:10.1371/journal.pone.0004709)
Supplement: Figure S1 — (0.18 MB DOC) [file pone.0004709.s002.doc]

**Stauch et al.**

**Targeting of Natural Killer cells by rabbit antithymocyte globulin and Campath-1H: similar effects independent of specificity**

**Figure S1**

**TNFα TNFα**

**Figure legend**

**Daclizumab and rIgG increase FasL, TNFα and IFNγ mRNA in NK cells. (A, B)** IL-2 (200IU/ml) pre-activated NK cells cultured in the presence of daclizumab and rIgG were analyzed for FASL, TNFα and IFNγ mRNA after 1, 2, 3 and 6 hours of co-culture. Both daclizumab and rIgG induced a rapid and dose-dependent induction of FasL, TNFα and IFNγ mRNA in NK cells which decreased after 6 hours of co-incubation. Values demonstrate the results relativized to untreated controls (2^-ΔΔct^) and are displayed as means of four independent experiments. Asterisks (*) indicate values that showed significantly higher induction compared to untreated controls; *p<0.05, ** p<0.01, *** p<0.001.
