# Supplementary material for: Targeting of Natural Killer Cells by Rabbit Antithymocyte Globulin and Campath-1H: Similar Effects Independent of Specificity
Source: PLoS One. 2009 Mar 5;4(3):e4709. doi: 10.1371/journal.pone.0004709 (PMC2651595; doi:10.1371/journal.pone.0004709)
Supplement: Figure S2 — (0.04 MB DOC) [file pone.0004709.s003.doc]

**Stauch et al.**

**Targeting of Natural Killer cells by rabbit antithymocyte globulin and Campath-1H: similar effects independent of specificity**

**Figure S2**

**Figure legend**

**CD16 ligation on NK cells is necessary for the induction of cytokine release by rATG and alemtuzumab.** IL2 (200IU/ml) pre-activated NK cells were treated for 1 hour either with 1µg/ml complete rATG or alemtuzumab or 1µg/ml F(ab) fragments of rATG or alemtuzumab. Additionally CD16 was blocked with F(ab) fragments (20µg/ml) of a blocking anti-CD16 mAb (3G8). After blocking CD16 NK cells were incubated with 1µg/ml rATG for 1 hour. Due to limited availability of the anti-CD16 antibody this experiment could not be performed for alemtuzumab. FasL, TNFα and IFNγ mRNA expression was assessed by real-time RT-PCR. The values gained were relativized against an untreated control. Rabbit ATG leads to a strong induction of FasL, TNFα and IFNγ mRNA which is prevented by a blockade of CD16. F(ab) fragments of rATG or alemtuzumab do not lead to an significant induction of FasL, TNFα or IFNγ mRNA, indicating the Fc-part as the active component of both antibodies. Values demonstrate the results related to untreated cells and are displayed as means of six independent experiments; p values are related to rATG or alemtuzumab treatment, *p<0.05, **p<0.01, ***p<0.001.
